# 1.55 Å resolution X-ray crystal structure of Rv3902c from *Mycobacterium tuberculosis*

**DOI:** 10.1107/S2053230X14003793

**Published:** 2014-03-25

**Authors:** Bharat G. Reddy, Derek B. Moates, Heung-Bok Kim, Todd J. Green, Chang-Yub Kim, Thomas C. Terwilliger, Lawrence J. DeLucas

**Affiliations:** a Department of Biochemistry and Molecular Genetics, University of Alabama at Birmingham, 1025 18th Street South, Birmingham, AL 35233, USA; b Department of Biology, University of Alabama at Birmingham, 1025 18th Street South, Birmingham, AL 35233, USA; c Bioscience Division, Los Alamos National Laboratory, Los Alamos, NM 87545, USA; d Department of Microbiology, University of Alabama at Birmingham, 1025 18th Street South, Birmingham, AL 35233, USA; e Department of Optometry, University of Alabama at Birmingham, 1025 18th Street South, Birmingham, AL 35233, USA

**Keywords:** *Mycobacterium tuberculosis*, Rv3902c, virulence-factor chaperone, novel fold

## Abstract

The crystallographic structure of the *Mycobacterium tuberculosis* (TB) protein Rv3902c (176 residues; molecular mass of 19.8 kDa) was determined at 1.55 Å resolution. The function of Rv3902c is unknown, although several TB genes involved in bacterial pathogenesis are expressed from the operon containing the Rv3902c gene. The unique structural fold of Rv3902c contains two domains, each consisting of antiparallel β-sheets and α-helices, creating a hand-like binding motif with a small binding pocket in the palm. Structural homology searches reveal that Rv3902c has an overall structure similar to that of the *Salmonella* virulence-factor chaperone InvB, with an r.m.s.d. for main-chain atoms of 2.3 Å along an aligned domain.

## Abstract 2

The 1.55 Å resolution X-ray crystal structure of Rv3902c from *M. tuberculosis* reveals a novel fold.

## 1. Introduction   

*Mycobacterium tuberculosis* (TB) is an aerobic acid-fast Gram-positive bacterium. A waxy coating on the cell surface of the TB organism makes it exceedingly resistant to host defense mechanisms, particularly when present in the alveoli of lung tissue. Despite the wide use of antibiotics and attenuated vaccines, TB is one of the leading causes of death from bacterial infections, killing an estimated 1.3 million people each year (World Health Organization, 2012 ▶). Today, the occurrence of multidrug-resistant tuberculosis (MDR-TB) and extensively drug-resistant tuberculosis (XRD-TB) is becoming more prevalent in both developing and industrialized nations, and has been reported in 84 countries (World Health Organization, 2012 ▶). There is a vital need for new vaccines and small-molecule therapeutics to combat these drug-resistant strains of TB. In 1998, researchers sequenced the complete genome of TB and revealed approximately 4000 genes (Cole *et al.*, 1998 ▶), which was followed by the establishment of the TB Structural Genomics Consortium under the NIH Protein Structure Initiative in 2000. The TB Structural Genomics Consortium is an effort to expedite the structure determination of all TB proteins in an effort to further the understanding of TB biology (Chim *et al.*, 2011 ▶). It is hoped that this structural knowledge will provide a basis for the identification of new protein targets and drug-discovery strategies with which to treat infections caused by TB.

Rv3902c is a TB protein with a molecular weight of 19.8 kDa comprised of 176 amino acids expressed on the same operon as EsxF, EsxE and Rv3903c (http://www.tbdb.org). The function of Rv3902c is unknown; however, the products of two genes transcribed along with Rv3902c, EsxE and EsxF, are paralogs to early secreted antigenic target 6 (Esat-6) proteins found in TB (Agarwal *et al.*, 2007 ▶). Esat-6 proteins are potent T-cell antigens and play a role in TB pathogenesis (Smith, 2003 ▶). Since Rv3902c is expressed on the same operon as Esat-6 proteins, this suggests that Rv3902c may play a role in TB virulence. Here, we present the crystal structure of the TB protein Rv3902c.

## 2. Materials and methods   

### 2.1. Protein expression and purification   

Rv3902c was subcloned into a pVP16 vector consisting of an N-­terminal fusion with a 6×His tag, maltose-binding protein (MBP) and a *Tobacco etch virus protease* (TEV) cleavage site *via* the Gateway cloning method (Invitrogen). It should be noted that owing to the TEV construct used, an additional serine remains at the N-­terminus of the protain after TEV cleavage. The resulting construct N-His_8_-MBP-linker-TEV+Rv3902c/pVP16 was transformed into *Escherichia coli* BL21(DE3) cells. A seed culture was created *via* the selection of a single ampicillin-resistant colony and inoculation into 25 ml Luria broth (LB) containing 100 µg ml^−1^ ampicillin (cultured overnight in an incubator shaker set at 250 rev min^−1^ and 37°C). On the following day, a 1:100 dilution of the seed culture was placed into two 1 l LB flasks each containing 100 µg ml^−1^ ampicillin and grown in an incubator/shaker set at 250 rev min^−1^ and 37°C for ∼4 h. When an OD_600_ of ∼0.6 was reached, the flasks were removed from the incubator, cooled on ice to 16°C, and isopropyl β-d-1-thiogalactopyranoside (IPTG) was added to a final concentration of 0.5 m*M* to induce expression. Induced cultures were allowed to grow overnight in an incubator/shaker set at 250 rev min^−1^ and 16°C.

The following morning, the incubated cells were centrifuged at 8000*g* and resuspended in 70 ml buffer *A* (20 m*M* Tris–HCl pH 8.0, 200 m*M* KCl, 5% glycerol, 1.4 m*M* β-mercaptoethanol) with 1× cOmplete Protease Inhibitor (Roche). All subsequent purification steps were performed either on ice or at 4°C. The cells were sonicated and the cellular debris was spun down at 25 000*g* for 30 min. The supernatant was filtered using a 0.45 µm filter, supplemented with imidazole to a final concentration of 20 m*M* and batch bound to 5 ml Ni–NTA Superflow beads (Qiagen) pre-equilibrated with buffer *A* and rocked for 1 h. The Ni–NTA beads were packed into a column and attached to an ÄKTA FPLC system (GE Healthcare). The Ni–NTA column was washed with five column volumes (CV) of 95% buffer *A* and 5% buffer *B* (buffer *A* plus 1 *M* imidazole) and eluted at 2 ml min^−1^ in a gradient from 5 to 100% buffer *B* over 10 CV. The purity of the peak fractions was analysed *via* SDS–PAGE followed by concentration of the peak fractions to a final volume of 5 ml with Amicon 10 000 NMWL (Millipore) spin concentrators. TEV protease was added to the pooled and concentrated fractions in a 1:100 (protease:protein) ratio to cleave the His-tagged MBP followed by a gentle overnight rocking. The sample was filtered with a 0.45 µm filter and applied onto a Superdex 75 size-exclusion column (26 mm diameter × 60 cm length; flow rate of 2.5 ml min^−1^) attached to an ÄKTA FPLC system pre-equilibrated with buffer *A*. The purity of the peak fractions was analyzed *via* SDS–PAGE. Since further purification was required to remove the His-tagged MBP, the peak fractions were applied onto a Ni–NTA column pre-equilibrated with buffer *A*. Because the purified and cleaved Rv3902c did not bind to the Ni–NTA column, the flowthrough was collected, concentrated to 25 mg ml^−1^ and then analysed *via* SDS–PAGE. The purified protein was snap-frozen in liquid nitrogen and stored at −80°C.

### 2.2. Crystallization, data collection and structure determination   

Nanocrystallization trials of purified Rv3902c were conducted with kits from Hampton Research, Emerald Bio and Qiagen. Each trial utilized a Gryphon (Art Robbins Instruments) nanodispensing robot with 200 nl drops (1:1 protein:well solution volume ratio) in 96-well sitting-drop plates. Several hits were found and optimized in 1 µl vapour-diffusion hanging drops (1:1 protein:well solution volume ratio), with the final optimized crystallization condition being 1.5 *M* ammonium sulfate, 200 m*M* sodium cacodylate pH 6.5. Clusters of hexagonal crystals often appeared 16–48 h later at room temperature. The crystals were manually separated with the Hampton Research Micro-Tools set, with some of the larger crystals reaching 0.2 × 0.2 × 0.5 mm.

Crystals were cryoprotected with a final concentration of 25% glycerol followed by flash-cooling in a nitrogen cryo-stream. Phases were obtained by soaking some of the crystals in cryosolution supplemented with 1 *M* sodium bromide for 30–60 s and immediately flash-cooling them. Diffraction data were collected on the SER-CAT 22-BM beamline at the Advanced Photon Source, Argonne National Laboratory. The bromide-soaked crystals were exposed to X-ray radiation (wavelength tuned to the bromide absorption edge at 0.9198 Å). A single-wavelength anomalous difference (SAD) data set was then collected to 1.62 Å resolution. Additionally, a native data set was collected at a wavelength of 1 Å to a resolution of 1.55 Å. Both data sets were indexed and scaled with *iMosflm* (Battye *et al.*, 2011 ▶) and *AIMLESS* (Winn *et al.*, 2011 ▶), respectively, and resolution limits were determined using the CC_1/2_ criterion (Karplus & Diederichs, 2012 ▶). The space group was determined to be *P*6_1_ using *POINTLESS* (Evans, 2006 ▶, 2011 ▶).

With one subunit in the asymmetric unit, the Matthews coefficient (*V*_M_) was calculated to be 3.32 Å^3^ Da^−1^, with an estimated solvent content of 63% (Kantardjieff & Rupp, 2003 ▶). The Br-atom positions, phasing and initial model were determined using the *AutoSol* module of *PHENIX* (Adams *et al.*, 2010 ▶). The model was further refined utilizing the native data set with iterative rounds of *PHENIX* automated refinement and manual refinement using *Coot* (Emsley & Cowtan, 2004 ▶). Residues 175–178 were not traced owing to missing electron density. Ramachandran plots revealed 98.3% of residues in favored regions and 1.7% in additionally allowed regions (Adams *et al.*, 2010 ▶). All structural figures, including the electrostatic map, were produced using *PyMOL* (http://www.pymol.org). The topology diagram was constructed with *Pro-origami* (Stivala *et al.*, 2011 ▶), with additional modifications made with *Inkscape* (http://inkscape.org/). Atomic coordinates and structure factors have been deposited in the Protein Data Bank (Berman *et al.*, 2004 ▶) with accession code 4o6g. Crystallographic data are summarized in Table 1 ▶.

## 3. Results and discussion   

### 3.1. General features of the Rv3902c structure   

**Figure 1:**
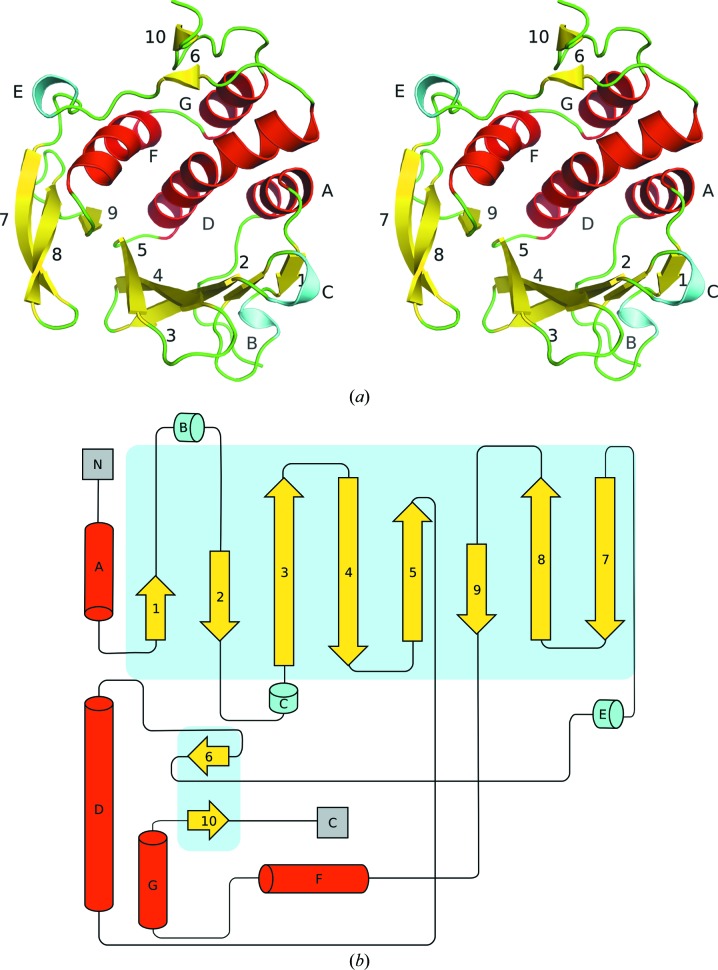
(*a*) Stereoview of the crystal structure of Rv3902c. (*b*) Secondary-structure diagram of Rv3902c. The structure is colored according to secondary-structure elements: loops, green; 3_10_-helices, cyan; α-helices, red; β-strands, yellow. Each β-strand (1–10) is numbered and each helix is lettered (A–G).

**Figure 2:**
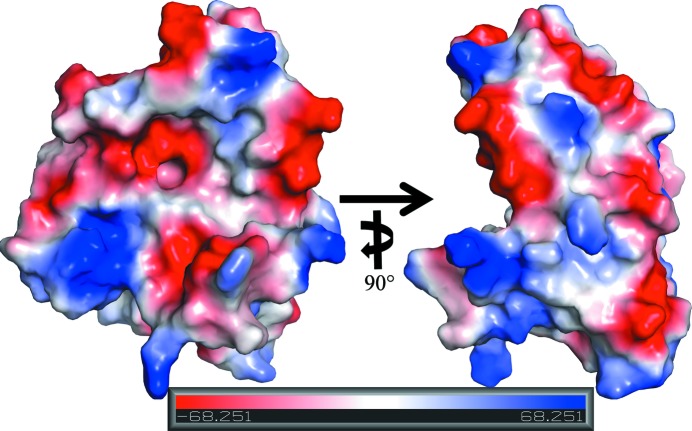
Surface electrostatic potential map of Rv3902c generated by *PyMOL*, with basic and acidic regions in blue and red, respectively. The views differ by a 90° rotation about the vertical axis.

Initial phases were determined *via* bromine-soaked crystals utilizing the single-wavelength anomalous dispersion (SAD) method (Dauter *et al.*, 2000 ▶). An additional high-resolution native crystal data set was collected to 1.55 Å resolution. The final structure consists of 174 residues and 216 waters (Fig. 1 ▶ *a*). Electron density was not observed for residues 175–178. The core of Rv3902c consists of two main structural domains. The first domain is composed of two antiparallel β-sheets containing β-strands 1–5 and 7–9 (Fig. 1 ▶ *b*) as well as two β–α–β motifs with 3_10_-helices (B and C) positioned between β-­strands 1 and 2 and between β-strands 2 and 3, and a third 3_10_ helix (E) immediately preceding β-strand 7. The second domain consists of α-helices A, D, F and G and two short antiparallel β-strands 6 and 10. A feature of notable functional interest is the creation of a hydrophobic pocket with an acidic entrance between α-helices D and F that is ∼7 Å in diameter and ∼7 Å deep (Fig. 2 ▶). The interior surface of this pocket is lined with the side chains of residues Tyr80, Tyr84, Leu145, Tyr148, Arg141 (the aliphatic portion) and Ile156 as well as the main chains of residues Lys79 and Gly83. The carboxylic acid moiety of Glu144 and the hydroxyl of Tyr148 are major acid charge contributors located at the entrance to the pocket. This small pocket is located in the center (palm) of a hand-like binding motif, with the bottom of the palm and thumbs made of the antiparallel β-strands 1–­5 and 3_10_-helices B and C, and with the fingers made up of α-helices D and F (Figs. 1 ▶ *a* and 2 ▶). The surface of Rv3902c is highly charged, with an estimated pI of 4.77 (Gasteiger *et al.*, 2005 ▶).

### 3.2. Homology search and analysis   

**Figure 3:**
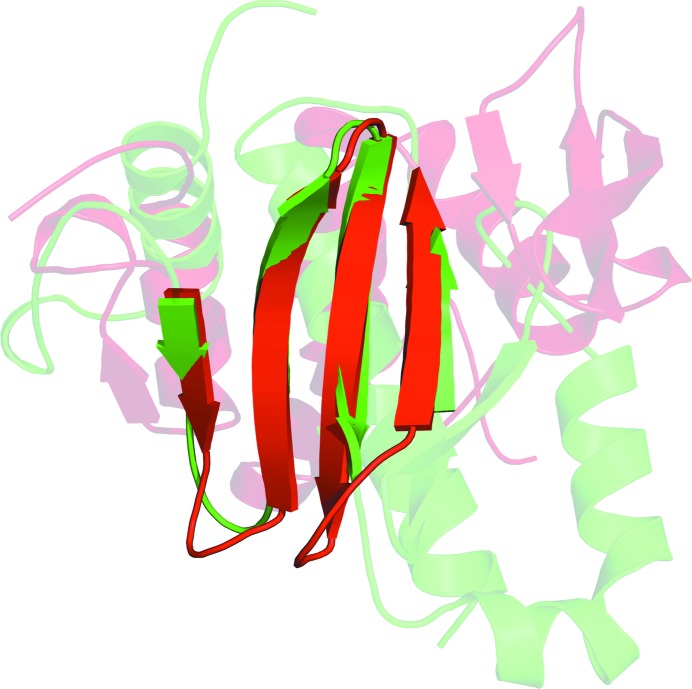
Alignment of Rv3902c (red) with InvB (green). The regions that are not transparent are the aligned homologous domains consisting of antiparallel β-­strands 2–5 of Rv3902c.

Three-dimensional structural homology searches utilizing the *iPBA* web server (Gelly *et al.*, 2011 ▶) did not identify structures with reasonable homology to Rv3902c. However, a type III virulence-factor chaperone, InvB, from *Salmonella* exhibited an r.m.s.d. of 2.3 Å along the aligned homologous domains comprising 24.2% of the main-chain structure (60 residues) of Rv3902c. Virulence-factor chaperones vary greatly in structural homology and sequence similarity and are typically small acidic proteins without an ATP-binding site or hydrolytic function that are involved in the secretion and translocation of bacterial virulence proteins (Lilic *et al.*, 2006 ▶). Rv3902c has some of the physical characteristics of these chaperones. Fig. 3 ▶ aligns the structures of Rv3902c and InvB along the aligned homologous domains. The aligned domain consists of the antiparallel β-strands 2–5 of Rv3902c, while much of the α-helical domains are not structurally homologous. If Rv3902c is a virulence-factor chaperone, it would represent the first known TB virulence-factor chaperone to be crystallized. Several other virulence-factor chaperones have been crystallized in other species and these virulence-factor chaperones are often in complex with their respective virulence factors (Lilic *et al.*, 2006 ▶; Phan *et al.*, 2004 ▶). The only other low-homology match to Rv3902c suggested by *iPBA* was a biotin protein ligase with a score of 3.2 Å along aligned regions comprising 10.5% of the structure of Rv3902c (Gelly *et al.*, 2011 ▶). An additional homology search was performed using *DALI *(Holm & Rosenström, 2010 ▶), but did not yield any clear matches among aligned domains. Finally, a functional search for Rv3902c conducted using the *ProFunc* server (Laskowski *et al.*, 2005 ▶) yielded no significant hits. We conclude that it is possible that there is a set of secreted virulence factors including EsxF, EsxE and Rv3903c, and that the Rv3902c protein may be a chaperone involved in the secretion of these proteins.

## Supplementary Material

PDB reference: Rv3902c, 4o6g

## Figures and Tables

**Table 1:** Summary of Rv3902c crystallographic data Values in parentheses are for the highest resolution shell.

	Native	Br-soaked
Experiment	Native	SAD
Wavelength ()	1.0000	0.9198
Unit-cell parameters ()	*a* = *b* = 91.81, *c* = 54.23	*a* = *b* = 92.08, *c* = 54.03
Space group	*P*6_1_	*P*6_1_
Resolution ()	39.761.55 (1.581.55)	32.081.62 (1.651.62)
No. of unique reflections	37938 (1875)	33300 (1646)
Completeness (%)	100 (100)	100 (100)
Multiplicity	14.7 (11.5)	7.5 (6.5)
Mean *I*/(*I*)	18.2 (2.1)	11.8 (2.0)
Molecules in asymmetric unit	1	1
Matthews coefficient (^3^Da^1^)	3.32	3.32
Solvent content (%)	62.90	62.98
*R*_merge_	0.087 (1.373)	0.096 (0.972)
*R*_meas_	0.094 (1.510)	0.110 (1.146)
*R*_p.i.m._	0.034 (0.617)	0.054 (0.603)
CC_1/2_	0.999 (0.609)	0.997 (0.597)
Anomalous completeness (%)		91.7 (88.8)
Anomalous multiplicity		3.5 (2.9)
Refinement statistics		
*R*_work_/*R*_free_ (%)	16.5/17.7	
No. of atoms		
Protein	1595	
Water	221	
Heavy atoms		18
R.m.s. deviations		
Bond lengths ()	0.006	
Bond angles ()	0.98	
Average *B* factor (^2^)		
Protein	22.1	
Water	37.3	
Ramachandran plot statistic, residues in		
Most favored regions	144	
Additionally allowed regions	8	
Generously allowed regions	0	
Disallowed regions	0	
*MolProbity* statistics		
Score	1.08	
Clashscore	2.95	
Poor rotamers (%)	0	
